# The Association Between Environmental and Social Factors and Myopia: A Review of Evidence From COVID-19 Pandemic

**DOI:** 10.3389/fpubh.2022.918182

**Published:** 2022-06-29

**Authors:** Jirawut Limwattanayingyong, Anyarak Amornpetchsathaporn, Methaphon Chainakul, Andrzej Grzybowski, Paisan Ruamviboonsuk

**Affiliations:** ^1^Department of Ophthalmology, Rajavithi Hospital, College of Medicine, Rangsit University, Bangkok, Thailand; ^2^Department of Ophthalmology, University of Warmia and Mazury, Olsztyn, Poland; ^3^Institute for Research in Ophthalmology, Foundation for Ophthalmology Development, Poznan, Poland

**Keywords:** myopia, COVID−19, lockdown, behavior, environment, social, association

## Abstract

**Purpose:**

To review the association between children's behavioral changes during the restriction due to the pandemic of Coronavirus disease (COVID-19) and the development and progression of myopia.

**Design:**

A literature review.

**Method:**

We looked for relevant studies related to 1) children's behavioral changes from COVID-19 restriction and 2) children's myopia progression during COVID-19 restriction by using the following keywords. They were “Behavior,” “Activity,” “COVID-19,” “Lockdown,” “Restriction,” and “Children” for the former; “Myopia,” “COVID-19,” “Lockdown,” “Restriction” for the latter. Titles, abstracts and full texts from the retrieved studies were screened and all relevant data were summarized, analyzed, and discussed.

**Results:**

Children were less active and more sedentary during COVID-19 restriction. According to five studies from China and six studies, each from Hong Kong, Spain, Israel, South Korea, Turkey and Taiwan included in our review, all countries without myopia preventive intervention supported the association between the lockdown and myopia progression by means of negative SER change ranging from 0.05–0.6 D, more negative SER change (compared post- to pre-lockdown) ranging from 0.71–0.98 D and more negative rate of SER changes (compared post- to pre-lockdown) ranging from 0.05–0.1 D/month. The reported factor that accelerated myopia is an increase in total near work, while increased outdoor activity is a protective factor against myopia progression.

**Conclusion:**

The pandemic of COVID-19 provided an unwanted opportunity to assess the effect of the behavioral changes and myopia in the real world. There is sufficient evidence to support the association between an increase in near work from home confinement or a reduction of outdoor activities and worsening of myopia during the COVID-19 lockdown. The findings from this review of data from the real world may help better understanding of myopia development and progression, which may lead to adjustment of behaviors to prevent myopia and its progression in the future.

## Introduction

Myopia or nearsightedness is a refractive error of spherical equivalent refraction (SER) ≤ −0.5 D (diopter), commonly developed in young children to early adolescence (1). It is a significant public and economic visual health problem worldwide. Deterioration of myopia can lead to many ocular complications and irreversible blindness (2, 3) The prevalence of myopia and high myopia (SER ≤ −5.00 D) (1) tend to increase year by year. By 2050, 4,758 million people (49.8% of the world population) and 938 million people (9.8% of the world population) are expected to have myopia and high myopia respectively (1).

Considering the factors influencing myopia development, three tiers of factors have been proposed by Seet B et al. (4) First, proximal or genetic factors, this hypothesis is supported by the evidence of higher number of myopic children in myopic parents than non-myopic parents (5, 6) and lower variations of SER and axial length (AL) in monozygotic twin than dizygotic twin.(7, 8) Second, intermediate or behavioral and environmental factors, there are evidences supporting that outdoor activity was an effective protective factor to control myopia prevalence, incidence, and also the prevention of myopia progression by slowing down the change of SER and minimizing AL elongation (9–11). On the other hand, the duration of near work was found to be significant higher in prevalent and incident myopes, compared to those with emmetropes of the same age (12, 13). Time spent on reading and other near work in childhood was also related to myopic progression in adulthood (14) although some studies did not find an evidence to support that near work was in correlation with myopia (15, 16). Third, distal or societal factors, there was evidence showed that students who ranked in the top quartile on educational performance in high myopia prevalence countries (Shanghai-China, Hong Kong-China, Taiwan, Singapore, Japan and South Korea) had higher engagement in after-school tutorial classes or cram schools than those with similar educational performance in low myopia prevalence countries (Australia, Canada, Finland). This trend of competitive and stressful education with cram schools in the East Asian countries might contribute to the higher prevalence of myopia (3, 17). Moreover, proportion of myopia tends to rise from outer suburban to inner urban areas and confine spaced residences, such as apartments, were also significantly associated with myopia (18).

The pandemic of coronavirus disease (COVID-19) due to severe acute respiratory syndrome coronavirus 2 (SARS-CoV-2) which originated in December 2019 in Wuhan, China, has caused a huge impact on health care of the entire world population. To mitigate the widespread of the disease, governments worldwide implemented the lockdown policy, including home confinement and temporary closure of public recreational and academic spaces (19). Children were forced to adapt to this circumstance by working and studying via online platforms instead of going to regular schools and doing any outdoor activities.

It is therefore plausible that these forced behavioral changes to the children due to the pandemic may have an impact on their refractive states, such as worsening of existing myopia or causing more new cases of myopia, during the lockdown period. This period provides an unwanted but relevant opportunity to find evidence of changes of myopia due to behavioral changes in the real world. The objective of this study is to review the evidence of the changes of myopia in children during the lockdown period due to the pandemic of COVID-19 in the literature.

## Method

We used electronic databases of PubMed, Medline and Google Scholar with the search terms or its combination of the following: “Behavior,” “Activity,” “COVID-19,” “Lockdown,” “Restriction,” “Children” to find 1) the evidences of children's behavioral changes, and the search terms or its combination of the following: “Myopia,” “COVID-19,” “Lockdown,” “Restriction” for 2) the evidences of changes of children's myopia condition during the COVID-19 lockdown. Associated references of these searched studies were included. Exclusion criteria for both topics were studies with irrelevant title or abstract and studies without their own original data.

For 1) the evidences of children's behavioral changes, we reviewed the abstracts of 1,924 studies. One thousand nine hundred and ten studies were excluded. The full articles of the remaining 14 studies were read. Studies were included if there were available full text in English and if they focused about general physical activities changes during the COVID-19 lockdown in general children or adolescents (not a specific group, for example medical students). Finally, nine studies were included in our review.

For 2) the evidences of changes of children's myopia condition during the COVID-19 lockdown, we reviewed the abstracts of 139 studies. We excluded the studies which measured myopia in subjective manner such as difficulty in seeing distant objects or eye strain. One hundred and twenty-three studies were excluded. The full article of the remaining 16 studies were read. Studies were included if there were available full text in English and if they reported an objective measurement of myopia prevalence and/or myopia incidence and/or myopia progression in general children or adolescents (not a specific group, for example medical students). Finally, eleven studies were included for review.

## Results

**Figure 1:**
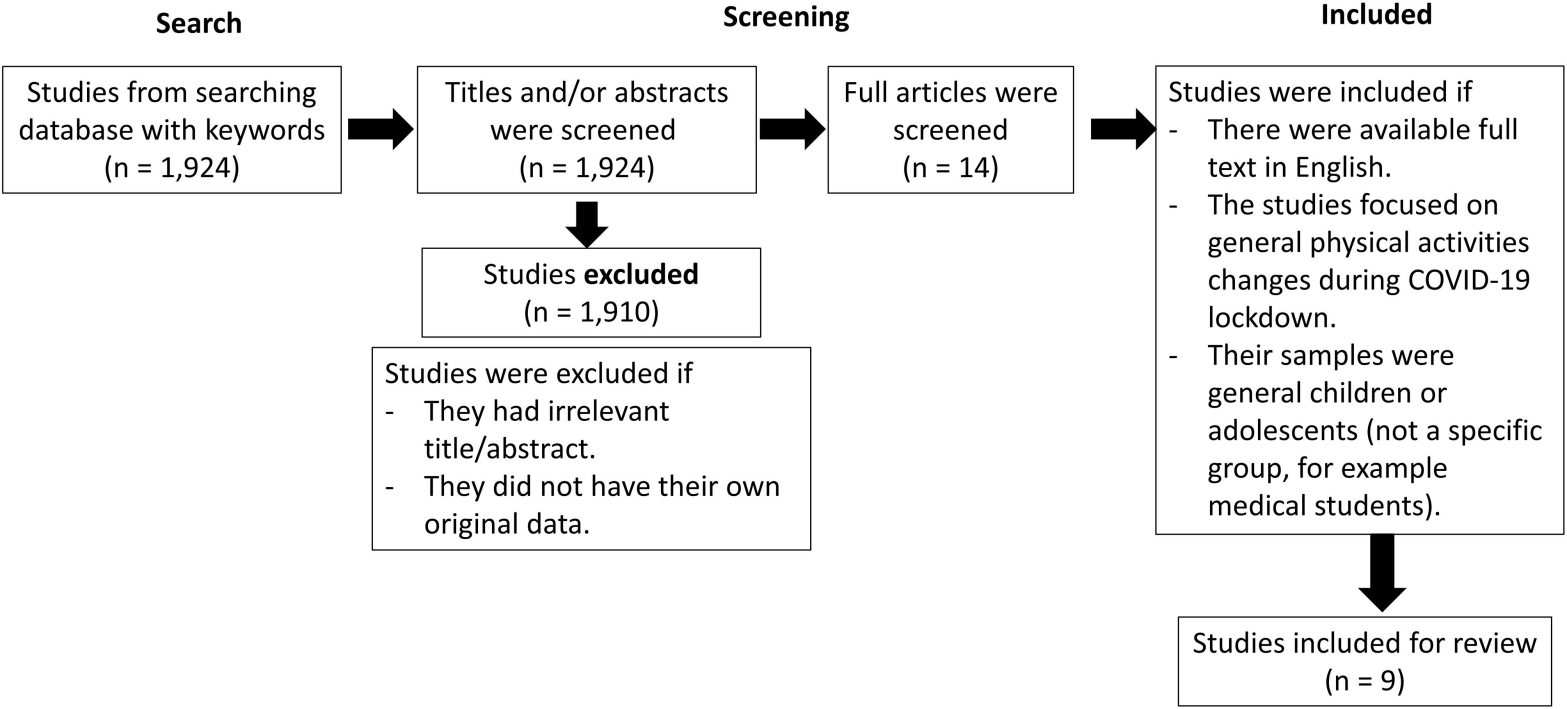
Search and selection strategies of children's behavioral changes during the COVID-19 lockdown.

Children's behavioral changes during the COVID-19 lockdown (Figure 1).

**Figure 2:**
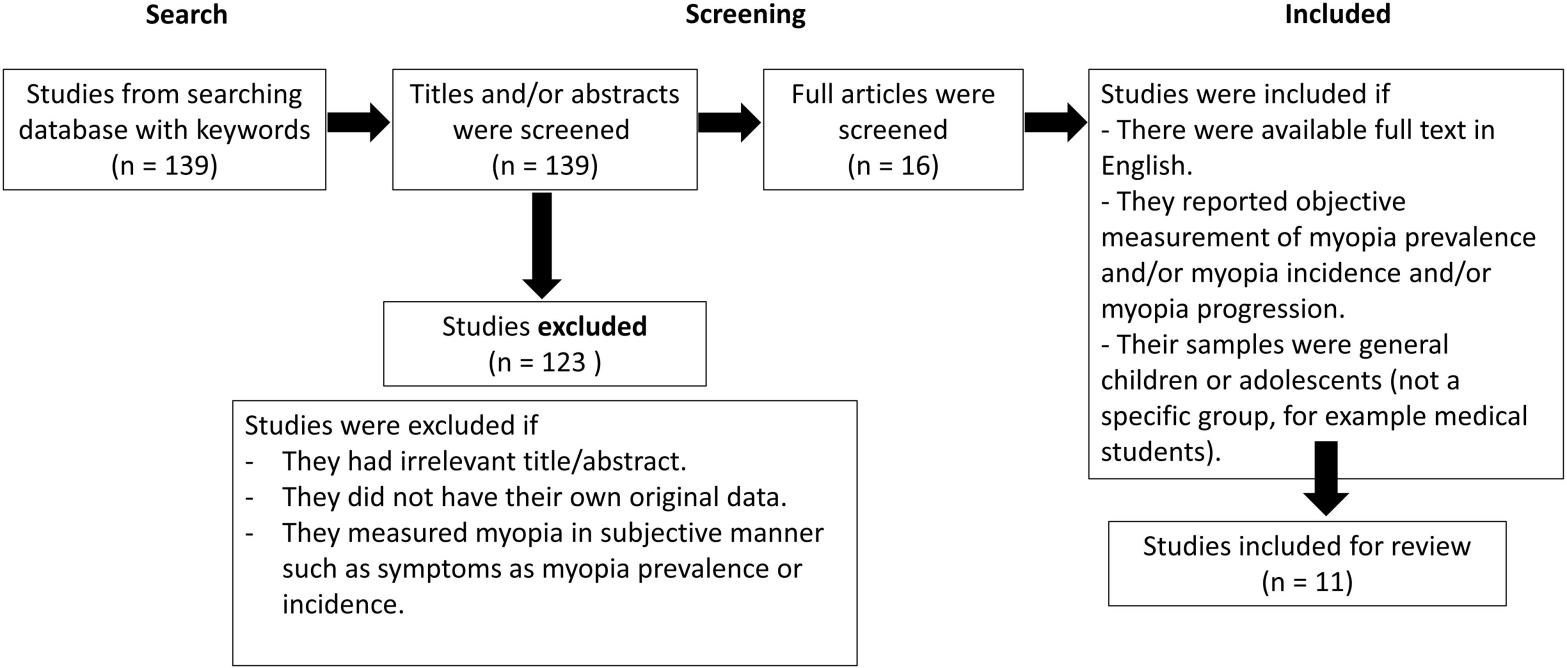
Search and selection strategies of the changes in myopia during the COVID-19 lockdown.

We reviewed nine studies from ten countries (Canada, USA, Spain, Brazil, Slovenia, the Netherlands, the United Kingdom [UK], India, China and Israel). All cross-sectional and longitudinal questionnaire-based studies consistently reported similar results: there is a reduction in time spent outdoor, such as for sports and physical activities, and an increase in sedentary time and digital screen time including social media uses during the COVID-19 lockdown (Outdoor time: decrease in outdoor time ranging from 5.4–7.25 h/week (20–22) or 5-point Likert type scale of 2.12/5 (1 = a lot less, 5 = a lot more) (23) or 19–47.5% of parents reported less physical activities of their children (24), Sedentary behavior: 28–47% of parents reported more sedentary behavior of their children (24), Screen time: increase in screen time ranging from 13.6–28.8 h/week (20, 22) or 5-point Likert type scale of 4.15/5 (1 = a lot less, 5 = a lot more)(23)). Two studies conducted in UK, Spain and Brazil also reported a significant decline in the number of children whose physical activities meet the World Health Organization (WHO) 24-h movement guidelines during the COVID-19 lockdown, compared to the period before the COVID-19 pandemic (decrease from 69.4% to 28.7% (25), from 3% to 0.3% and 11.7% to 7.5% in Spanish and Brazilian respectively (26)). In addition, researchers from Israel and the Netherlands also reported a reduction in physical and outdoor activities using an objective measuring method of accelerometry in pre and during COVID-19 lockdown (decrease physical activities from 1236 CPM to 1003 CPM, *p* < 0.019 (27), from 595 CPM to 429 CPM, *p* = 0.001(28) and decrease outdoor time from 1.8 h/day to 0.7 h/day, *p* = 0.01 (28)). These results were comparable with those results based on subjective questionnaire-based

method described previously.The changes in myopia during the COVID-19 lockdown (Figure 2).

We included eleven studies (five studies from China and six studies, each from Hong Kong, Taiwan, South Korea, Israel, Turkey and Spain). The studies were conducted in either of the two settings: school-based setting or clinical-based setting. Cycloplegia was used in some studies to assess children's myopia status. The study from Taiwan is the only study conducted in the situation where myopia preventive intervention was implemented.

The characteristic and measurement outcomes of the selected studies without myopia preventive intervention are demonstrated in Table 1 and the characteristic and measurement outcomes of the selected studies with myopia preventive intervention are demonstrated in Table 2.

**Table 1:** The selected studies without myopia preventive interventions, their characteristics and measurement outcomes.

				**Measurement outcomes**			
**Setting**	**Myopic status assessment**	**Study [country, participant (N)]**	**Study design**	**Prevalence (%)** **(Age, Pre vs. Post lockdown)**	**Incidence (%) (Age, Pre vs. Post lockdown)**	**SER (Diopter)**	**AL elongatio*n* (mm)**
School–based setting	Non–cycloplegic photorefraction	Wang J et al. (29) (China, 123,535)	Longitudinal cohort	6, 5.7 vs. 21.5 7, 16.2 vs. 26.2 8, 27.7 vs. 37.2 9–13, minimal change	NA	6, −0.32 7, −0.28 8, −0.29 9, −0.12 10, −0.11 11, −0.06 12, −0.05 13, −0.05^*^	NA
	Non–cycloplegic autorefraction	Chang P et al. (30) (China, 44,187)	Longitudinal cohort	NA, 53.2 vs. 73.7	NA	−0.030, −0.074, 0.016 ø	NA
	Cycloplegic autorefraction	Hu Y et al. (31) (China, 2,114)	Cross–sectional cohorts	Grade3, 13.3 vs. 20.8	Grade3, 7.5 vs.15.3	Grade3, −0.36^*^ (*p* < 0.01)	Grade3, 0.08‡ (*p* < 0.01)
		Zhang X et al. (32) (Hong Kong, 1,793)	Cross–sectional cohorts	NA	Cannot compare ^†^	6, −0.54 7, −0.53 8, −0.44 6–8, −0.5^*^ (*p* < 0.001)	6, 0.3 7, 0.31 8, 0.26 6–8, 0.29‡ (*p* < 0.001)
Clinical–based setting	Non–cycloplegic autorefraction	Peregrina C et al. (33) (Spain, 1,600)	Longitudinal cohort	NA	NA	5, −0.21 (*p* = 0.005) 6, −0.05 (*p* = 0.078) 7, −0.26 (*p* = 0.008) 5–7, −0.18^*^ (p ≤ 0.001)	NA
	Cycloplegic autorefraction	Ma D et al. (34) (China, 291)	Cross–sectional cohorts	NA	NA	8–10, −0.60D^**^ (*p* < 0.001)	8–10, 0.01‡ (*p* = 0.37)
		Ma M et al. (35) (China, 201)	Cross–sectional cohorts	NA	NA	7–12, −0.39 vs. −0.98¶ (*p* < 0.001)	NA
		Erdinest N et al. (36) (Israel, 14)	Longitudinal cohort	NA	NA	9–15, −0.33 vs.−0.74¶¶ (*p* < 0.001)	9–15, 0.29 vs. 0.47‡‡
		Yum H et al. (37) (South Korea, 103)	Longitudinal cohort	NA	NA	5–7, −0.066 vs. −0.103 (*p* = 0.028) 8–10, −0.044 vs. −0.064 (*p* = 0.002) 11–15, −0.038 vs. −0.049 ϕϕ (*p* = 0.065)	5–7, 0.036 vs. 0.05 (*p* = 0.022) 8–10, 0.024 vs. 0.03 (*p* = 0.005) 11–15, 0.017 vs. 0.017‡‡‡ (*p* = 0.792)
		Aslan F et al. (38) (Turkey, 115)	Longitudinal cohort	NA	NA	8–17, −0.54 vs. −0.71 ¶¶ (*p* = 0.003)	NA

*Longitudinal cohort means samples were the same group of children but different ages. Cross–sectional cohorts mean samples were in the different groups of children but the same age*.

† *Cannot compare because not equal follow–up time in each sub study (3 years in pre–covid cohort vs. 8 months in covid cohort)*.

* *Age, mean diopter change from pre to post lockdown*.

** *Age, mean diopter change from pre to during lockdown*.

*¶Age, mean diopter change pre vs. during lockdown*.

*¶¶Age, mean diopter change pre vs. post lockdown*.

*øRate of diopter change per month in pre, during, post– lockdown*.

*ø øAge, rate of diopter change per month in pre vs. post lockdown*.

*‡Age, mean AL change from pre to during lockdown*.

*‡‡Age, mean AL change in pre vs. post lockdown*.

*‡‡‡Age, mean AL change per month in pre vs. post lockdown*.

**Table 2:** The selected study with myopia preventive interventions, its characteristics and measurement outcomes.

				**Measurement outcomes**			
**Study approach**	**Myopic status assessment**	**Study [country, participant (*N)*]**	**Study design**	**Prevalence (%)** **(Age, Range between pre & post lockdown)**	**Incidence (%) (Age, Pre vs. Post lockdown)**	**SER (Diopter)**	**AL elongation (mm)**
Population–based approach	Cycloplegic autorefraction	Yang Y et al. (39) (Taiwan, 23,930)	Longitudinal cohort	5–6, 8.5% – 10.3%	NA	NA	NA

*Longitudinal cohort means samples were the same group of children but different ages*.

We classified the selected studies into two types in this review. A total of seven studies were conducted in cohorts of the same group of children at different ages (Longitudinal cohort), the other four studies were conducted in cohorts of the different groups of children at the same age (Cross-sectional cohorts). All studies without myopia preventive intervention found at least one of the following outcomes: increased myopia prevalence (ranging from 7.5–20.5% with a trend of more rising of prevalence in younger age groups (29)), increased myopia incidence (from 7.5% to 15.3%), greater myopia progression (negative SER change ranging from 0.05–0.98 D (29, 31–36, 38) or negative rate of SER changes ranging from 0.05-0.1 D/month (30, 37)) during the COVID-19 lockdown. With preventive interventions, a study in Taiwan reported stable myopia prevalence throughout the COVID-19 lockdown (39).

A total of five studies monitored the changes in axial length (AL) during the COVID-19 lockdown. (31, 32, 34, 36, 37) Two of these studies reported an increase in AL during the lockdown (ranging from 0.08–0.31 mm) (31, 32). Another two studies found faster AL elongation during the lockdown (0.47 mm/year compared to 0.29 mm/year (36), the rate of AL increase ranging from 0.03–0.05 mm/month (37)). Whereas the only study from China (34) reported no difference of AL before and after that lockdown period (0.01 mm, *p* = 0.37).

Behavioral changes during the lockdown were evaluated by a questionnaire in seven studies. (32–35, 37–39) Increase in time spending on total near work, including reading, homework, online learning and digital screen time (2.4–4.63 h/day) (32, 34, 35, 37) and a reduction in total outdoor activities (0.1–0.86 h/day) (32, 34, 35, 37) were reported.

Among these studies, four studies reported the direct association of specific behavioral changes and worsening of myopia. Two studies from China (34, 35) found that an increase of digital screen time was associated with a greater SER change (odds ratio 2.658, 95% CI 1.587 to 4.450, *p* < 0.005(34) and 0.211, 95%CI 0.280 to 0.142, *p* < 0.001(35) respectively). Moreover, online education was also found to be associated with a greater SER change in Chinese children (odds ratio 3.717, 95% CI 1.587 to 8.665, *p* = 0.02) (34). Hongkong specified that increase in reading time was associated with myopic shift in term of a greater change of SER (odds ratio −0.04, 95% CI −0.07 to −0.01, *p* = 0.02) and an AL elongation (odds ratio 0.03, 95% CI 0.01 to 0.05, *p* = 0.01) (32). And 2-year exposure to myopia preventive intervention was found to be a protective factor against myopia progression among Taiwanese children (odds ratio, 0.56, 95% CI, 0.50 to 0.63, *p* < 0.001) (39).

## Discussion

We found indirect evidence showing the association between children's forced decrease in outdoor activities and increase in near work and worsening of myopia during the lockdown due to COVID-19. This finding is relevant in both settings we found in the literature. For the school-based settings, most studies were conducted in the high myopic prevalence countries where the eye examination and refraction are annually screened in each school. The advantages of the school-based approach are a large sample size and a completeness of the refractive data of the targeted population of children. On the contrary, for the clinical-based setting, most studies were conducted in the lower myopic prevalence region. The refractive data of the children in this setting are from those who regularly visit eye clinics in each year and may not represent the real-world population.

To obtain children's refractive status, both non-cycloplegic and cycloplegic refraction were used in the reviewed studies. Non-cycloplegic auto/photorefraction are easier and faster to conduct but their results are known to be exaggerated (40). This method is useful in the school-based screening where the cycloplegia may not properly be conducted. On the other hand, cycloplegic autorefraction, which requires more steps to instill a drop of cycloplegic medication into the children's eyes, is more reliable and commonly used in the clinical setting. Despite these different refractive methods, the study results on changes in myopia during the lockdown were similar.

For the studies on the cohorts of children in the same group at the different ages (Longitudinal cohort), the refractive data was collected in a group of samples at least three different time points: before the pandemic, at the beginning of the pandemic (before the lockdown) and after the pandemic (after the lockdown). This means that the refractive data of the same group of the children was analyzed and compared in a temporal relation. It is well documented that there is a progression of myopia among children in their natural history (per year). However, the progression of myopia reported in these studies were higher in terms of diopter changes and faster compared with the natural history.

For the studies on the cohorts of different groups of children at the same age (Cross-sectional cohorts), the refractive data between these two groups of the children, a group before the pandemic and another group during the pandemic, were compared. This study design eliminated the confounding effect of normal myopia progression with increasing age and might reflect the true effect of the social restriction on myopia progression. China is a country where there has been a high concern about the high prevalence of myopia. Half of the studies in this review were conducted in China, three of them were school-based (29–31) whereas the other two were clinical setting studies (34, 35). The detailed analysis in some of these studies indicated that specific types of near work might have an influence on myopic progression. Children who used mobile phones had the fastest myopia progression, followed by those who used tablets. Projectors and televisions might be better choices for online learning since both of them associated with significantly less myopic shift, compared to the other devices (35).

Apart from China, the school-based refraction screening was also conducted with related databases existing in Hong Kong and Taiwan. Taiwan (39) is the only country with myopia preventive intervention in this review. Interestingly, the study in Taiwan was the only study that showed no significant changes in myopic prevalence during the lockdown. It is possible that the reason behind this success was the Yilan Myopia Prevention and Vision Improvement Program (YMVIP), the initiative launched by the Taiwan government to promote children's outdoor activities. This policy was successfully implemented in 2014 and the prevalence of myopia continuously decreased in an L-shaped pattern since then. During the lockdown, Taiwanese were encouraged but not enforced to absolute stay at home, therefore, the promoting intervention may not be disrupted and children could continue to spend their time outdoors.

There were two clinical-based studies that observed that myopia could still progress during the lockdown even in children with myopia who were treated with atropine. This was from the review of medical records of 103 children in South Korean (37) and 14 children in Israel (36)). Even under the effect of atropine treatment, a greater SER progression (from 0.33D/year to 0.74 D/year, *p* < 0.001) and a greater AL elongation (from 0.21 mm/year to 0.47 mm/year, *p* < 0.001) between pre- and post-COVID-19 were found significantly in all Israeli children in the study. The faster rate of SER progression (from 0.047 D/month to 0.067 D/month, *p* < 0.001) and AL elongation (from 0.024 mm/month to 0.030 mm/month, *p* = 0.001) between pre- and post-COVID-19 were also found in South Korean children aged 5 to 7 and 8 to 10 years who were still under the treatment with atropine, but no statistically significant changes were observed in older children aged 11 to 15 years.

The age effect was also observed in some studies (29) in which a greater shift of myopic was observed in children aged 6–8 years than those who were older than 8 years old.

The worsening of myopia found during the COVID-19 lockdown suggested that even temporary increase in near work or decrease in outdoor activities for about 3 months can induce this condition. There are two main theories that may be used for the explanation. First, a defocusing theory (41). Defocusing means blurring of perceived images due to an accommodative lag. In response to the blurred vision, there is a release of chemicals causing AL elongation as a compensation for defocusing. This structural change of eye balls can be permanent. The second theory is about accommodative spasm or near work induced transient myopia (NITM). NITM is caused by the remaining accommodative effect after an abrupt change from a long duration of near work to distant vision. Hence, it can be reversed after a period of time (42).

From our review, there were five studies (31, 32, 34, 36, 37) evaluating AL before and after the pandemic of COVID-19. Four out of five studies (31, 32, 36, 37) found that AL elongation was faster during the lockdown. These findings might support the defocusing theory. Another study (34), meanwhile, did not find a statistical difference in AL between pre and post home studying. These two mechanisms of myopia might also be found in combination, since partial reversible of myopia progression was found after the lockdown was over in a study (30). A temporary accommodative spasm might be accounted for in the reversed part of myopia while the remaining was from permanent AL elongation.

The main strength of our review is the inclusion of studies from many countries in which the settings were both school-based and clinical-based. Therefore, the populations that we reviewed may represent a large number of children from many ethnicities. Limitations include difficulty to compare results from various studies. For example, refractive data from studies using non-cycloplegic measurement might overestimate the SER than those with cycloplegic measurements. Moreover, the refractive data after termination of the lockdown may be required to assess the permanent effect of myopic shift.

## Conclusion

The pandemic of COVID-19 provided an unwanted opportunity to assess the effect of the behavioral changes and myopia in the real world. There is sufficient evidence to support the association between an increase in near work from home confinement or a reduction of outdoor activities and worsening of myopia during the COVID-19 lockdown. This worsening was found even in children who were under treatment with atropine. On the other hand, an initiative to increase outdoor activities, such as the Yilan Myopia Prevention and Vision Improvement Program in Taiwan, may be able to stabilize myopia progression during the lockdown period. The findings from this review of data from the real world may help better understanding of myopia development and progression, which may lead to adjustment of behaviors to prevent myopia and its progression in the future.

## Author Contributions

Substantial contributions to the conception or design of the work, or the acquisition, analysis, or interpretation of data for the work: JL, AA, MC, AG, and PR. Drafting the work or revising it critically for important intellectual content: JL, AA, MC, and PR. Final approval of the version to be published: JL, AG, and PR. All authors contributed to the article and approved the submitted version.

## Funding

This project was supported by Rajavithi research grant number 059/2565.

## Conflict of Interest

The authors declare that the research was conducted in the absence of any commercial or financial relationships that could be construed as a potential conflict of interest.

## Publisher's Note

All claims expressed in this article are solely those of the authors and do not necessarily represent those of their affiliated organizations, or those of the publisher, the editors and the reviewers. Any product that may be evaluated in this article, or claim that may be made by its manufacturer, is not guaranteed or endorsed by the publisher.

## References

[1] Holden BA Fricke TR Wilson DA Jong M Naidoo KS Sankaridurg P. Global Prevalence of Myopia and High Myopia and Temporal Trends from 2000 through 2050. Ophthalmology. (2016) 123:1036-42. 10.1016/j.ophtha.2016.01.006 26875007

[2] Saw SM. How blinding is pathological myopia? Br J Ophthalmol. (2006) 90:525–6. 10.1136/bjo.2005.087999 16622078 PMC1857043

[3] Haarman AEG Enthoven CA Tideman JWL Tedja MS Verhoeven VJM Klaver CCW. The complications of myopia: a review and meta-analysis. Invest Ophthalmol Vis Sci. (2020) 61:49. 10.1167/iovs.61.4.49 32347918 PMC7401976

[4] Seet B Wong TY Tan DT Saw SM Balakrishnan V Lee LK. Myopia in Singapore: taking a public health approach. Br J Ophthalmol. (2001) 85:521–6. 10.1136/bjo.85.5.521 11316705 PMC1723957

[5] Zhang X Qu X Zhou X. Association between parental myopia and the risk of myopia in a child. Exp Ther Med. (2015) 9:2420–8. 10.3892/etm.2015.2415 26136998 PMC4473431

[6] Jiang D Lin H Li C Liu L Xiao H Lin Y. Longitudinal association between myopia and parental myopia and outdoor time among students in Wenzhou: a 2. 5-year longitudinal cohort study. BMC Ophthalmol. (2021) 21:11. 10.1186/s12886-020-01763-9 33407251 PMC7789164

[7] Teikari JM O'Donnell J Kaprio J Koskenvuo M. Impact of heredity in myopia. Hum Hered. (1991) 41:151–6. 10.1159/000153994 1937488

[8] Dirani M Chamberlain M Shekar SN Islam AF Garoufalis P Chen CY. Heritability of refractive error and ocular biometrics: the Genes in Myopia (GEM) twin study. Invest Ophthalmol Vis Sci. (2006) 47:4756–61. 10.1167/iovs.06-0270 17065484

[9] Sherwin JC Reacher MH Keogh RH Khawaja AP Mackey DA Foster PJ. The association between time spent outdoors and myopia in children and adolescents: a systematic review and meta-analysis. Ophthalmology. (2012) 119:2141–51. 10.1016/j.ophtha.2012.04.020 22809757

[10] Xiong S Sankaridurg P Naduvilath T Zang J Zou H Zhu J. Time spent in outdoor activities in relation to myopia prevention and control: a meta-analysis and systematic review. Acta Ophthalmol. (2017) 95:551–66. 10.1111/aos.13403 28251836 PMC5599950

[11] Cao K Wan Y Yusufu M Wang N. Significance of outdoor time for myopia prevention: a systematic review and meta-analysis based on randomized controlled trials. Ophthalmic Res. (2020) 63:97–105. 10.1159/000501937 31430758

[12] Huang HM Chang DS Wu PC. The Association between near work activities and myopia in children-a systematic review and meta-analysis. PLoS ONE. (2015) 10:e0140419. 10.1371/journal.pone.0140419 26485393 PMC4618477

[13] French AN Morgan IG Mitchell P Rose KA. Risk factors for incident myopia in Australian schoolchildren: the Sydney adolescent vascular and eye study. Ophthalmology. (2013) 120:2100–8. 10.1016/j.ophtha.2013.02.035 23672971

[14] Pärssinen O Kauppinen M Viljanen A. The progression of myopia from its onset at age 8-12 to adulthood and the influence of heredity and external factors on myopic progression a 23-year follow-up study. Acta Ophthalmol. (2014) 92:730–9. 10.1111/aos.12387 24674576

[15] Huang L Kawasaki H Liu Y Wang Z. The prevalence of myopia and the factors associated with it among university students in Nanjing: a cross-sectional study. Medicine (Baltimore). (2019) 98:e14777. 10.1097/MD.0000000000014777 30855486 PMC6417623

[16] Lu B Congdon N Liu X Choi K Lam DS Zhang M. Associations between near work, outdoor activity, and myopia among adolescent students in rural China: the Xichang Pediatric Refractive Error Study report no 2. Arch Ophthalmol. (2009) 127:769–75. 10.1001/archophthalmol.2009.105 19506196

[17] Morgan IG Rose KA. Myopia and international educational performance. Ophthalmic Physiol Opt. (2013) 33:329–38. 10.1111/opo.12040 23662964

[18] Ip JM Rose KA Morgan IG Burlutsky G Mitchell P. Myopia and the urban environment: findings in a sample of 12-year-old Australian school children. Invest Ophthalmol Vis Sci. (2008) 49:3858–63. 10.1167/iovs.07-1451 18469186

[19] Fund IM. Policy Responses to COVID-19 2021. Available online at: https://www.imf.org/en/Topics/imf-and-covid19/Policy-Responses-to-COVID-19#C (accessed February 11, 2022).

[20] Saxena R Gupta V Rakheja V Dhiman R Bhardawaj A Vashist P. Lifestyle modification in school-going children before and after COVID-19 lockdown. Indian J Ophthalmol. (2021) 69:3623–9. 10.4103/ijo.IJO_2096_21 34827007 PMC8837368

[21] Morrison SA Meh K Sember V Starc G Jurak G. The effect of pandemic movement restriction policies on children's physical fitness, activity, screen time, and sleep. Front Public Health. (2021) 9:785679. 10.3389/fpubh.2021.785679 34938712 PMC8685208

[22] Xiang M Zhang Z Kuwahara K. Impact of COVID-19 pandemic on children and adolescents' lifestyle behavior larger than expected. Prog Cardiovasc Dis. (2020) 63:531–2. 10.1016/j.pcad.2020.04.013 32360513 PMC7190470

[23] Moore SA Faulkner G Rhodes RE Brussoni M Chulak-Bozzer T Ferguson LJ. Impact of the COVID-19 virus outbreak on movement and play behaviours of Canadian children and youth: a national survey. Int J Behav Nutr Phys Act. (2020) 17:85. 10.1186/s12966-020-00987-8 32631350 PMC7336091

[24] Dunton GF Do B Wang SD. Early effects of the COVID-19 pandemic on physical activity and sedentary behavior in children living in the U. S BMC Public Health. (2020) 20:1351. 10.1186/s12889-020-09429-3 32887592 PMC7472405

[25] Bingham DD Daly-Smith A Hall J Seims A Dogra SA Fairclough SJ. Covid-19 lockdown: Ethnic differences in children's self-reported physical activity and the importance of leaving the home environment; a longitudinal and cross-sectional study from the Born in Bradford birth cohort study. Int J Behav Nutr Phys Act. (2021) 18:117. 10.1186/s12966-021-01183-y 34488785 PMC8419665

[26] López-Gil JF Tremblay MS Brazo-Sayavera J. Changes in healthy behaviors and meeting 24-h movement guidelines in spanish and brazilian preschoolers, children and adolescents during the COVID-19 lockdown. Children (Basel). (2021) 8:83. 10.3390/children8020083 33530315 PMC7912043

[27] Ten Velde G Lubrecht J Arayess L van Loo C Hesselink M Reijnders D. Physical activity behaviour and screen time in Dutch children during the COVID-19 pandemic: Pre-, during- and post-school closures. Pediatr Obes. (2021) 16:e12779. 10.1111/ijpo.12779 33624443 PMC7995017

[28] Shneor E Doron R Levine J Zimmerman DR Benoit JS Ostrin LA. Objective Behavioral Measures in Children before, during, and after the COVID-19 Lockdown in Israel. Int J Environ Res Public Health. (2021) 18:8732. 10.3390/ijerph18168732 34444483 PMC8394769

[29] Wang J Li Y Musch DC Wei N Qi X Ding G. Progression of Myopia in school-aged children after COVID-19 home confinement. JAMA Ophthalmol. (2021) 139:293–300. 10.1001/jamaophthalmol.2020.6239 33443542 PMC7809617

[30] Chang P Zhang B Lin L Chen R Chen S Zhao Y. Comparison of Myopic Progression before, during, and after COVID-19 Lockdown. Ophthalmology. (2021) 128:1655–7. 10.1016/j.ophtha.2021.03.029 33771516 PMC7986471

[31] Hu Y Zhao F Ding X Zhang S Li Z Guo Y. Rates of Myopia Development in Young Chinese Schoolchildren During the Outbreak of COVID-19. JAMA Ophthalmol. (2021) 139:1115–21. 10.1001/jamaophthalmol.2021.3563 34529002 PMC8446907

[32] Zhang X Cheung SSL Chan HN Zhang Y Wang YM Yip BH. Myopia incidence and lifestyle changes among school children during the COVID-19 pandemic: a population-based prospective study. Br J Ophthalmol. (2021). 10.1136/bjophthalmol-2021-319307 34340973

[33] Alvarez-Peregrina C Martinez-Perez C Villa-Collar C Andreu-Vázquez C Ruiz-Pomeda A Sánchez-Tena M. Impact of COVID-19 home confinement in children's refractive errors. Int J Environ Res Public Health. (2021) 18:5347. 10.3390/ijerph18105347 34067888 PMC8156137

[34] Ma D Wei S Li SM Yang X Cao K Hu J. Progression of myopia in a natural cohort of Chinese children during COVID-19 pandemic. Graefes Arch Clin Exp Ophthalmol. (2021) 259:2813–20. 10.1007/s00417-021-05305-x 34287693 PMC8294263

[35] Ma M Xiong S Zhao S Zheng Z Sun T Li C. COVID-19 Home Quarantine Accelerated the Progression of Myopia in Children Aged 7 to 12 Years in China. Invest Ophthalmol Vis Sci. (2021) 62:37. 10.1167/iovs.62.10.37 34463719 PMC8411864

[36] Erdinest N London N Levinger N Lavy I Pras E Morad Y. Decreased effectiveness of 0.01% atropine treatment for myopia control during prolonged COVID-19 lockdowns. Cont Lens Anterior Eye. (2021):101475. 10.1016/j.clae.2021.101475 34238687 PMC9278875

[37] Yum HR Park SH Shin SY. Influence of coronavirus disease 2019 on myopic progression in children treated with low-concentration atropine. PLoS ONE. (2021) 16:e0257480. 10.1371/journal.pone.0257480 34520481 PMC8439482

[38] Aslan F Sahinoglu-Keskek N. The effect of home education on myopia progression in children during the COVID-19 pandemic. Eye (Lond). (2021): 1-6. 10.1038/s41433-021-01655-2. [Epub ahead of print]. PMC8243061 34193982

[39] Yang YC Hsu NW Wang CY Shyong MP Tsai DC. Prevalence trend of Myopia after promoting eye care in preschoolers: a serial survey in Taiwan before and during the Coronavirus Disease 2019 pandemic. Ophthalmology. (2022) 129:181–90. 10.1016/j.ophtha.2021.08.013 34425129

[40] Sankaridurg P He X Naduvilath T Lv M Ho A Smith E. 3rd. Comparison of noncycloplegic and cycloplegic autorefraction in categorizing refractive error data in children. Acta Ophthalmol. (2017) 95:e633–e40. 10.1111/aos.13569 29110438 PMC5698763

[41] Day M DL. Myopia and defocus: the current understanding. Scandinavian J Optom Vis Sci. (2011) 4:1–14. 10.5384/sjovs.vol1i4p1-14

[42] Vasudevan B Ciuffreda KJ Gilmartin B. Sympathetic inhibition of accommodation after sustained nearwork in subjects with myopia and emmetropia. Invest Ophthalmol Vis Sci. (2009) 50:114–20. 10.1167/iovs.08-1762 18599570

